# Association between sex hormones and erectile dysfunction in men without hypoandrogenism

**DOI:** 10.1038/s41598-024-64339-3

**Published:** 2024-06-11

**Authors:** Naoki Fujita, Teppei Okamoto, Hayato Yamamoto, Takahiro Yoneyama, Yasuhiro Hashimoto, Tatsuya Mikami, Ken Itoh, Chikara Ohyama, Shingo Hatakeyama

**Affiliations:** 1 Department of Urology, Hirosaki University Graduate School of Medicine, 5-Zaifucho, Hirosaki, 036-8562 Japan; 2 Department of Advanced Blood Purification Therapy, Hirosaki University Graduate School of Medicine, Hirosaki, 036-8562 Japan; 3 Innovation Center for Health Promotion, Hirosaki University Graduate School of Medicine, Hirosaki, 036-8562 Japan; 4 Department of Stress Response Science, Center for Advanced Medical Science, Hirosaki University Graduate School of Medicine, Hirosaki, 036-8562 Japan

**Keywords:** Erectile dysfunction, Sexual dysfunction, Hypogonadism

## Abstract

In addition to testosterone, various endocrine hormones, such as dehydroepiandrosterone sulfate (DHEA-S) and estradiol, may be involved in erectile function. However, the role of these sex hormones in the erectile function of men without hypoandrogenism remains unclear. This cross-sectional study included 398 community-dwelling men without hypoandrogenism. The participants were categorized into the non-ED and ED groups. Multivariable logistic regression analyses were performed to investigate the relationship between ED and serum sex hormone levels, including total testosterone, DHEA-S, estradiol, luteinizing hormone (LH), follicle-stimulating hormone (FSH), and prolactin. Among the 398 men, 66 (17%) and 332 (83%) were categorized into the non-ED and ED groups, respectively. In the multivariable analyses, serum DHEA-S and estradiol levels were significantly associated with ED (odds ratio [OR]: 0.996, *P* = 0.030; OR: 1.082, *P* = 0.002; respectively), whereas serum total testosterone, LH, FSH, and prolactin levels did not demonstrate significant association. After adjusting for age, none of neutrophil-to-lymphocyte ratio, serum plasminogen activator inhibitor-1 levels, and skin advanced glycation end-products levels demonstrated significant correlation with serum DHEA-S and estradiol levels. In conclusion, lower testosterone levels did not affect ED in men with normal testosterone levels, whereas serum DHEA-S and estradiol levels were significantly associated with ED.

## Introduction

Testosterone regulates almost all components involved in erectile function, from the pelvic ganglions to endothelial and smooth muscle cells of the corpora cavernosa^1,2^. Hypogonadism, defined as testosterone deficiency with associated symptoms, is linked to erectile dysfunction (ED) through intricate pathogenic mechanisms^3^ and is now well recognized as a risk factor for ED^4^. However, the role of testosterone in the erectile function of men without hypoandrogenism remains unclear.

In addition to testosterone, various endocrine hormones may be involved in erectile function. Dehydroepiandrosterone sulfate (DHEA-S) is the most abundant steroid hormone in humans^5^, which is reported to play a multifunctional protective role in cellular well-being^6,7^. The Massachusetts Male Aging Study (MMAS) had revealed that out of 17 investigated hormones only DHEA-S exhibited significant correlation with ED^8^. Additionally, estradiol and prolactin have been reported to affect erectile function^9,10^. However, several conflicting results have prevented us from reaching definitive conclusions^11^. Moreover, the role of these sex hormones in the erectile function of men with normal testosterone levels remains unclear.

In the present study, first, we evaluated the relationship between sex hormone levels and ED in community-dwelling men without hypoandrogenism. Second, we investigated the correlation between DHEA-S and estradiol levels and well-known risk factors for ED, including low-grade systemic inflammation, endothelial dysfunction, and advanced glycation end-products (AGEs) accumulation.

## Methods

### Ethics statement

This study was conducted in accordance with the principles of the Declaration of Helsinki. Data on community-dwelling men were collected from the Iwaki Health Promotion Project, and the study was approved by the Ethics Review Board of the Hirosaki University Graduate School of Medicine (authorization number: 2022–097). All the participants provided written informed consent.

### Participant selection

**Figure 1:**
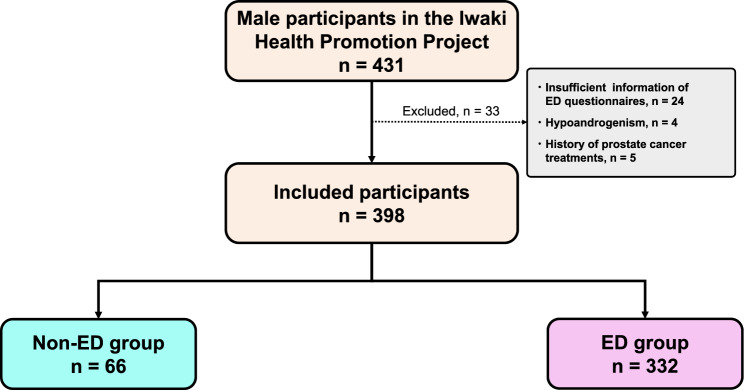
Selection of the study participants. Number of included and excluded participants. *ED* erectile dysfunction.

Among the 431 male participants in the Iwaki Health Promotion Project 2015, 33 were excluded based on the following exclusion criteria: (1) insufficient information on erectile function, (2) hypoandrogenism, and (3) history of prostate cancer treatment, which affect erectile function. Consequently, the final analysis included 398 community-dwelling men without hypoandrogenism (Fig. 1).

### Evaluation of variables

The following variables were analyzed: age, body mass index, hypertension (HTN), dyslipidemia, diabetes mellitus (DM), smoking status, current habitual drinking, educational level, medications (β-blockers, calcium [Ca]-blockers, and thiazide diuretics), mental health status, laboratory values, ankle-brachial index (ABI), and brachial-ankle pulse wave velocity (baPWV). Blood samples were collected in the morning. Serum total testosterone, estradiol, luteinizing hormone (LH), follicle-stimulating hormone (FSH), and prolactin levels were measured using a chemiluminescent immunoassay. Serum DHEA-S level was measured using a chemiluminescent enzyme immunoassay. Hypoandrogenism was defined as a serum total testosterone level ≤ 250 ng/dL^6^. The accumulation of skin AGEs can be assessed non-invasively by measuring skin autofluorescence as previously described^6^.

### Evaluation of ED

ED was assessed using the 5 item International Index of Erectile Function (IIEF-5). The participants were categorized into the non-ED (IIEF-5 score ≥ 22) and ED (IIEF-5 score ≤ 21) groups (Fig. 1).

### Statistical analysis

Differences in quantitative variables between the two groups were analyzed using the Mann–Whitney U test. Categorical variables were compared using the Fisher’s exact test or chi-square test. Correlations between variables were analyzed using Spearman’s rank correlation coefficients. Univariable and multivariable logistic regression analyses were performed to identify significant factors associated with ED. Due to the high correlation among sex hormones and relatively limited number of events in comparison to the sample size in the present study, we included sex hormones in the multivariable regression models separately. Consequently, six multivariable regression analyses were performed. As low-grade systemic inflammation, endothelial dysfunction, and AGEs accumulation are well-known risk factors for ED^12,13^, multiple linear regression analyses were performed with serum DHEA-S and estradiol levels as dependent variables and age, neutrophil-to-lymphocyte ratio (NLR), serum von Willebrand factor (vWF) and plasminogen activator inhibitor-1 (PAI-1) levels, and skin AGEs levels as independent variables.

## Results

### Background of participants

The median age of the study participants and their IIEF-5 scores were 53 years and 17, respectively. Among the 398 men, 66 (17%) and 332 (83%) were classified into the non-ED and ED groups, respectively (Fig. 1).

Age, prevalence of HTN, dyslipidemia, and DM, educational level, number of Ca-blocker users, renal function, and baPWV values were significantly different between the two groups (Table 1).

**Table 1:** Background of participants.

	All n = 398	Non-ED group n = 66	ED group n = 332	*P* value
Age, years	53 (38–65)	36 (32–44)	57 (43–66)	< 0.001
Body mass index, kg/m^2^	23 (22–26)	23 (21–25)	23 (22–26)	0.759
Hypertension	167 (42%)	13 (20%)	154 (46%)	< 0.001
Dyslipidemia	128 (32%)	14 (21%)	114 (34%)	0.037
Diabetes mellitus	41 (10%)	2 (3.0%)	39 (12%)	0.043
Smoking status				0.448
Never	154 (39%)	30 (45%)	124 (37%)	
Former	130 (33%)	20 (30%)	110 (33%)	
Current	114 (29%)	16 (24%)	98 (30%)	
Current habitual drinking	268 (67%)	44 (67%)	224 (68%)	0.899
Post-high school education	108 (27%)	27 (41%)	81 (24%)	0.006
Medications				
β-blockers	10 (2.5%)	1 (1.5%)	9 (2.7%)	1.000
Calcium-blockers	45 (11%)	2 (3.0%)	43 (13%)	0.018
Thiazide diuretics	10 (2.5%)	0 (0.0%)	10 (3.0%)	0.380
SF-36 MCS	51 (45–56)	49 (44–56)	51 (45–56)	0.298
Laboratory blood test				
eGFR, mL/min/1.73m^2^	81 (72–89)	84 (76–94)	81 (71–88)	0.013
Total cholesterol, mg/dL	203 (179–225)	201 (174–219)	205 (180–225)	0.176
LDL-cholesterol, mg/dL	116 (95–132)	111 (90–123)	116 (96–134)	0.055
Triglyceride, mg/dL	97 (68–148)	98 (54–162)	97 (71–147)	0.351
Ankle-brachial index	1.1 (1.1–1.2)	1.1 (1.1–1.2)	1.1 (1.1–1.2)	0.271
baPWV	1460 (1278–1721)	1293 (1185–1418)	1499 (1314–1791)	< 0.001
IIEF-5 score	17 (12–20)	23 (22–24)	15 (11–19)	< 0.001

All data are presented as n (%) or medians (interquartile ranges).

*ED* erectile dysfunction, *SF-36 MCS* 36-Item Short Form Health Survey Mental Component Summary, *eGFR* estimated glomerular filtration rate, *LDL* low density lipoprotein, *baPWV* brachial-ankle pulse wave velocity, *IIEF* International Index of Erectile Function.

### Correlations between serum sex hormone levels and IIEF-5 scores

**Figure 2:**
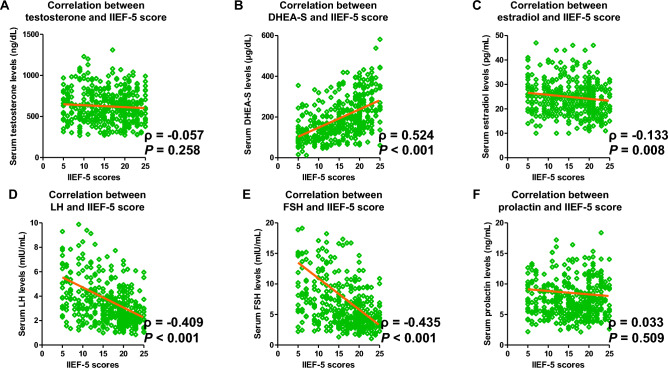
Correlations between serum sex hormone levels and the 5 item International Index of Erectile Function (IIEF-5) scores. Correlations between serum sex hormone levels and IIEF-5 scores have been analyzed using Spearman’s rank correlation coefficient (**A**–**F**). *DHEA-S* dehydroepiandrosterone sulfate, *LH* luteinizing hormone, *FSH* follicle-stimulating hormone.

Spearman’s rank correlation test revealed that serum total testosterone and prolactin levels did not exhibit a significant correlation with IIEF-5 scores (Fig. 2A,F; ρ =  − 0.057, *P* = 0.258; ρ = 0.033, *P* = 0.509; respectively). Serum DHEA-S levels were positively and significantly correlated with IIEF-5 scores (Fig. 2B; ρ = 0.524, *P* < 0.001), whereas serum estradiol, LH, and FSH levels were negatively and significantly correlated with IIEF-5 scores (Fig. 2C–E; ρ =  − 0.133, *P* = 0.008; ρ =  − 0.409, *P* < 0.001; and ρ =  − 0.435, *P* < 0.001; respectively).

### Associations between serum sex hormone levels and ED

**Figure 3:**
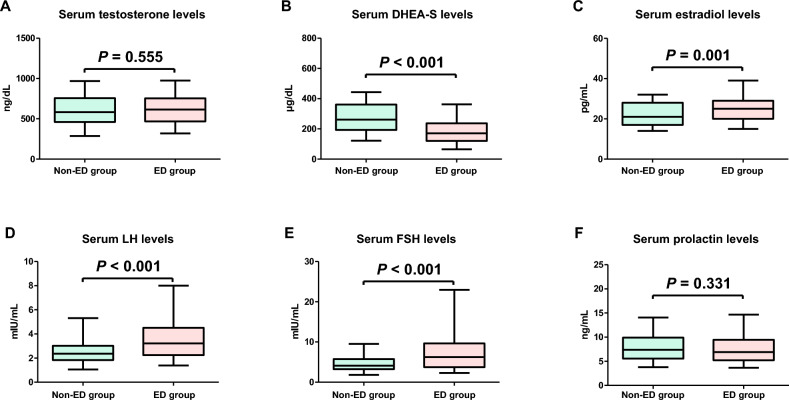
Differences of serum sex hormone levels between non-erectile dysfunction (ED) and ED groups. Serum sex hormone levels are compared between the two groups using the Mann–Whitney U test (**A**–**F**). *DHEA-S* dehydroepiandrosterone sulfate, *LH* luteinizing hormone, *FSH* follicle-stimulating hormone.

**Figure 4:**
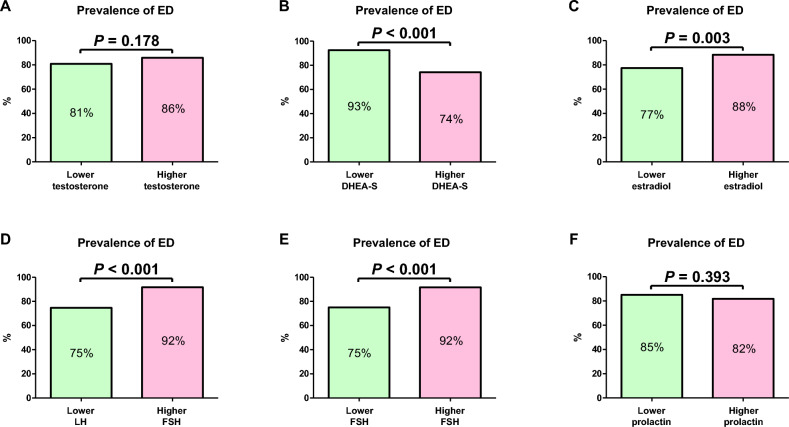
Prevalence of erectile dysfunction (ED). Men are categorized according to the median sex hormone levels. The prevalence of ED is compared between men with lower sex hormone levels and those with higher sex hormone levels using the chi-square test (**A**–**F**). *DHEA-S* dehydroepiandrosterone sulfate, *LH* luteinizing hormone, *FSH* follicle-stimulating hormone.

Serum total testosterone and prolactin levels were not significantly different between the non-ED and ED groups (Fig. 3A,F; *P* = 0.555 and *P* = 0.331, respectively). Serum DHEA-S levels in the ED group were significantly lower than those in the non-ED group (Fig. 3B; *P* < 0.001), whereas serum estradiol, LH, and FSH levels in the ED group were significantly higher than those in the non-ED group (Fig. 3C–E; *P* = 0.001, *P* < 0.001, and* P* < 0.001, respectively). The median serum testosterone, DHEA-S, estradiol, LH, FSH, and prolactin levels were 608 ng/dL, 184 µg/dL, 24 pg/mL, 3.0 mIU/mL, 5.6 mIU/mL, and 7.0 ng/mL, respectively. When men were categorized into groups based on their median sex hormone levels, no significant differences in the prevalence of ED were observed between men with higher and lower testosterone and prolactin levels (Fig. 4A,F; *P* = 0.178 and *P* = 0.393, respectively). In contrast, the prevalence of ED in men with lower DHEA-S and higher estradiol, LH, and FSH levels was significantly higher than that in men with higher DHEA-S and lower estradiol, LH, and FSH levels (Fig. 4B–E; *P* < 0.001, *P* = 0.003, *P* < 0.001, and* P* < 0.001, respectively).

### Univariable and multivariable analyses for ED

In the univariable analyses, age, HTN, dyslipidemia, eGFR, and baPWV were significantly associated with ED (Table S1). In the multivariable analyses adjusted for these confounding variables, serum DHEA-S and estradiol levels were significantly associated with ED (Table 2; odds ratio [OR]: 0.996, *P* = 0.030; OR: 1.082, *P* = 0.002; respectively), whereas serum total testosterone, LH, FSH, and prolactin levels did not demonstrate significant association (Table 2).

**Table 2:** Multivariable analyses for erectile dysfunction.

	Factor	*P* value	Odds ratio	95% CI
Total testosterone*	Continuous	0.167	1.001	1.000–1.003
DHEA-S*	Continuous	0.030	0.996	0.992–1.000
Estradiol*	Continuous	0.002	1.082	1.030–1.136
LH*	Continuous	0.415	1.108	0.865–1.420
FSH*	Continuous	0.542	1.034	0.929–1.151
Prolactin*	Continuous	0.420	1.028	0.962–1.098

*CI* confidence interval, *DHEA-S* dehydroepiandrosterone sulfate, *LH* luteinizing hormone, *FSH* follicle-stimulating hormone.

*Adjusted for age, hypertension, dyslipidemia, diabetes mellitus, estimated glomerular filtration rate, and brachial-ankle pulse wave velocity.

### Correlations of serum DHEA-S and estradiol levels with systemic inflammatory and endothelial dysfunction markers and skin AGEs levels

In the multiple linear regression analyses, after adjusting for age, none of NLR, serum vWF and PAI-1 levels, and skin AGEs levels did not demonstrate significant correlation with serum DHEA-S levels (Table 3) and serum estradiol levels (Table 4).

**Table 3:** Multiple linear regression analyses for serum dehydroepiandrosterone sulfate levels.

	Partial regression coefficient (B)	Standard error	Standardized partial regression coefficient (β)	t value	*P* value
Constant	419.3	14.72		28.49	< 0.001
Age	− 4.357	0.252	− 0.668	− 17.27	< 0.001
**NLR**	5.473	5.204	0.041	1.052	0.294
Constant	430.1	14.29		30.10	< 0.001
Age	− 4.156	0.282	− 0.638	− 14.72	< 0.001
**vWF**	− 0.095	0.096	− 0.043	− 0.983	0.326
Constant	416.1	15.26		27.26	< 0.001
Age	− 4.264	0.248	− 0.654	− 17.20	< 0.001
**PAI-1**	0.214	0.169	0.048	1.266	0.206
Constant	405.8	17.81		22.78	< 0.001
Age	− 4.568	0.322	− 0.703	− 14.21	< 0.001
**AGEs**	17.90	10.92	0.081	1.640	0.102

*NLR* neutrophil-to-lymphocyte ratio, *vWF* von Willebrand factor, *PAI-1* plasminogen activator inhibitor-1, *AGEs* advanced glycation end-products.

**Table 4:** Multiple linear regression analyses for serum estradiol levels.

	Partial regression coefficient (B)	Standard error	Standardized partial regression coefficient (β)	t value	*P* value
Constant	21.99	1.485		14.81	< 0.001
Age	0.049	0.025	0.099	1.935	0.054
**NLR**	0.122	0.524	0.012	0.233	0.816
Constant	21.45	1.426		15.04	< 0.001
Age	0.022	0.028	0.045	0.797	0.426
**vWF**	0.017	0.010	0.100	1.756	0.080
Constant	21.94	1.530		14.34	< 0.001
Age	0.047	0.025	0.096	1.908	0.057
**PAI-1**	0.008	0.017	0.025	0.496	0.620
Constant	22.73	1.797		12.65	< 0.001
Age	0.049	0.032	0.099	1.524	0.128
**AGEs**	− 0.238	1.091	− 0.014	− 0.218	0.827

*NLR* neutrophil-to-lymphocyte ratio, *vWF* von Willebrand factor, *PAI-1* plasminogen activator inhibitor-1, *AGEs* advanced glycation end-products.

## Discussion

To the best of our knowledge, this is the first study to evaluate the association between sex hormone levels and ED in community-dwelling men without hypoandrogenism. The study results indicated that testosterone had no impact on ED in men without hypoandrogenism; however serum DHEA-S and estradiol levels demonstrated independent and significant association with ED. Nevertheless, well-established risk factors for ED, including low-grade systemic inflammation, endothelial dysfunction, and AGEs accumulation, did not exhibit any correlation with serum DHEA-S and estradiol levels. These findings suggested the involvement of unknown mechanisms for development of ED and indicated the need for further studies to clarify the biological mechanisms underlying the effects of DHEA-S and estradiol on ED.

Although hypoandrogenism is a well-known risk factor for ED^4^, serum total testosterone levels were not associated with ED in men with normal testosterone levels in the present study. This result is consistent with those of previous studies. Rhoden et al. conducted a cross-sectional study in a large consecutive series including approximately 1,000 older men and demonstrated no significant correlation between serum total testosterone levels and IIEF-5 scores (*P* = 0.612)^14^. Similarly, the MMAS conducted by Feldman et al. revealed that none of the total testosterone, free testosterone, or albumin-bound testosterone levels exhibited significant correlation with ED^8^. However, the reasons for these negative results remain unclear. Endothelial dysfunction is believed to be one of the mechanisms by which hypoandrogenism causes ED. In hypoandrogenism animal models, testosterone was observed to regulate nitric oxide formation by acting on endothelial and neuronal nitric oxide synthases^15,16^. Additional our analysis revealed that serum testosterone levels exhibited negative and significant correlation with serum PAI-1 levels, a well-known endothelial dysfunction marker, after adjusting for age, HTN, and DM (Table S2). In the present study, regardless of the significant correlation between serum testosterone levels and endothelial dysfunction, lower testosterone levels had no effect on ED. Since the pathogenic mechanisms linking low testosterone levels with ED are complex^15^, further studies are necessary to elucidate the role of testosterone in erectile function in men with normal testosterone levels.

In the present study, serum DHEA-S levels were significantly associated with ED in men without hypoandrogenism. Although several studies have supported this finding, none have exclusively examined men without hypoandrogenism^8,17,18^. Since DHEA has its own receptors on vascular endothelial cells^19^, both DHEA and DHEA-S have various biological functions besides being precursors of testosterone and estradiol^20^. DHEA has been reported to activate potassium channels via the activation of soluble guanylate cyclase and trigger nitric oxide synthesis through G-protein-dependent activation and stabilization of endothelial nitric oxide synthase, independent of androgen receptors^21–23^. Besides its effect as a modulator of endothelial function, DHEA has a multifunctional protective effect in many aspects of cellular well-being^6,7^, including the improvement of insulin sensitivity, reduction of fibrinolysis suppressor, and antiatherosclerotic and antioxidative effects^24–26^. However, the present study failed to demonstrate a significant correlation between DHEA-S levels and well-known risk factors for ED, including systemic inflammation, endothelial dysfunction, and AGEs accumulation (Table 3). Because its precise physiological function remains unknown, further research is required to establish a link between erectile function and DHEA-S in men without hypoandrogenism.

Despite several studies investigating the association between ED and estradiol, the results have been inconclusive due to conflicting results and small sample sizes. In the present study, serum estradiol levels exhibited significant association with ED. This result is consistent with that of a previous meta-analysis, including 1,249 patients with ED and 1,270 healthy individuals^9^. However, the biological mechanisms that link ED with estradiol remain unclear. Although its antagonistic effect on testosterone through the sympathetic and parasympathetic nervous systems is thought to be one of the mechanisms^27^, testosterone had no effect on ED in the present study. Since estradiol receptors are abundantly expressed in penile tissues^28^, estradiol has been reported to directly impact the erectile function, alongside its effects mediated by testosterone, including an increase in venous vascular permeability via vascular endothelial growth factor, impairment of the corpus cavernosum relaxation, and changes in the structure of the corpus cavernosum^9,29,30^. Although these mechanisms might support our results, drawing a definitive conclusion is difficult owing to several limitations of the present study. Further prospective studies are warranted to elucidate the effects of estradiol on ED in men without hypoandrogenism.

This study had several limitations. First, its cross-sectional nature prevented us from determining cause-and-effect associations. Second, this study included a relatively small number of men with normal erectile function. Third, because the level of sexual inactivity of Japanese men is high^31^, a lack of information on the sexual activity might cause an underestimation of erectile function using the IIEF-5.

In conclusion, testosterone had no effect on ED in men with normal testosterone levels, whereas serum DHEA-S and estradiol levels were significantly associated with ED. These results broaden our understanding of the etiology of ED in men without hypoandrogenism.

### Supplementary Information

Supplementary Table S1.

Supplementary Table S2.

## Data Availability

The data that support the findings of this study are available from the Iwaki Health Promotion Project but restrictions apply to the availability of these data, which were used under license for the current study, and so are not publicly available. Data are however available from the authors upon reasonable request and with permission of the Iwaki Health Promotion Project.
