# Supplementary material for: Association between sex hormones and erectile dysfunction in men without hypoandrogenism
Source: Sci Rep. 2024 Jun 11;14:13433. doi: 10.1038/s41598-024-64339-3 (PMC11167061; doi:10.1038/s41598-024-64339-3)
Supplement: Supplementary file 1 — Supplementary Table S1. [file 41598_2024_64339_MOESM1_ESM.docx]

**Table S1 Univariable analyses for erectile dysfunction**

|  | Factor | *P* value | Odds ratio | 95% CI |
| --- | --- | --- | --- | --- |
| Age | Continuous | <0.001 | 1.086 | 1.061–1.112 |
| Hypertension | Present | <0.001 | 3.527 | 1.853–6.715 |
| Dyslipidemia | Present | 0.040 | 1.942 | 1.032–3.655 |
| Diabetes mellitus | Present | 0.050 | 4.259 | 1.003–18.10 |
| Smoking status | Current | 0.388 | 1.309 | 0.711–2.410 |
| Current habitual drinking | Positive | 0.899 | 1.037 | 0.592–1.817 |
| eGFR | Continuous | 0.008 | 0.974 | 0.956–0.993 |
| baPWV | Continuous | <0.001 | 1.003 | 1.002–1.004 |
| Total testosterone | Continuous | 0.491 | 1.000 | 0.999–1.002 |
| DHEA-S | Continuous | <0.001 | 0.991 | 0.989–0.994 |
| Estradiol | Continuous | 0.001 | 1.074 | 1.028–1.121 |
| LH | Continuous | <0.001 | 1.615 | 1.289–2.023 |
| FSH | Continuous | <0.001 | 1.255 | 1.129–1.395 |
| Prolactin | Continuous | 0.785 | 1.004 | 0.973–1.036 |

CI, confidence interval; eGFR, estimated glomerular filtration rate; baPWV, brachial-ankle pulse wave velocity; DHEA-S, dehydroepiandrosterone-sulfate; LH, luteinizing hormone; FSH, follicle-stimulating hormone.
