# Supplementary material for: Association between sex hormones and erectile dysfunction in men without hypoandrogenism
Source: Sci Rep. 2024 Jun 11;14:13433. doi: 10.1038/s41598-024-64339-3 (PMC11167061; doi:10.1038/s41598-024-64339-3)
Supplement: Supplementary file 2 — Supplementary Table S2. [file 41598_2024_64339_MOESM2_ESM.docx]

**Table S2 Multiple linear regression analyses for serum plasminogen activator inhibitor-1 levels**

|  | Partial regression coefficient (B) | Standard error | Standardized  partial regression coefficient (β) | t value | *P* value |
| --- | --- | --- | --- | --- | --- |
| Constant | 58.57 | 5.240 |  | 11.18 | <0.001 |
| Age | -0.267 | 0.079 | -0.182 | -3.379 | 0.001 |
| Hypertension | 6.613 | 2.514 | 0.145 | 2.631 | 0.009 |
| Diabetes mellitus | 9.289 | 3.788 | 0.126 | 2.452 | 0.015 |
| Testosterone | -0.019 | 0.006 | -0.165 | -3.331 | 0.001 |
